# Effects of maternal nonylphenol exposure on the proliferation of glial cells in the brain of male offspring mice

**DOI:** 10.1080/19768354.2024.2401389

**Published:** 2024-09-12

**Authors:** Seung Hyun Lee, Hyun Seung Shin, Yun Hee So, Dong Hun Lee, Jin Yeop Kim, Eun-Hee Lee, Eui-Man Jung

**Affiliations:** a Department of Molecular Biology, College of Natural Sciences, Pusan National University, Busan, Republic of Korea; b Institute for Future Earth, Pusan National University, Busan, Republic of Korea; c BIOLINKs Inc., Chuncheon, Republic of Korea; d Department of Microbiology, College of Natural Sciences, Pusan National University, Busan, Republic of Korea

**Keywords:** Endocrine disrupting chemicals, Nonylphenol, Glial cells, Brain development

## Abstract

Glial cells play a significant role in maintaining brain homeostasis and normal brain development, and their functions can be impaired by exposure to endocrine disruptors. 4-n-Nonylphenol (NP), a representative endocrine disruptor, is widely used in personal care products and industrial materials. NP accumulates in various organs, including the brain, of living organisms and adversely influences brain health. However, studies on the effects of NP on glial cells are limited. This study aims to investigate the effects of NP on glial cells using primary mixed glial cells and offspring mice exposed to NP during gestation and lactation. *In vitro* experiments revealed that NP exposure stimulated the astrocytes and microglia proliferation but not oligodendrocytes. NP exposure activated microglia and reduced myelin protein expression in oligodendrocytes. Moreover, maternal NP exposure increased the numbers of microglia and oligodendrocytes in the cerebral cortex of adult offspring. NP exposure caused anxiety– and depressive-like behaviors in adult mice. Collectively, these findings suggest that maternal NP exposure negatively affects the brain development in adult offspring mice.

## GRAPHICAL ABSTRACT

**Figure:**
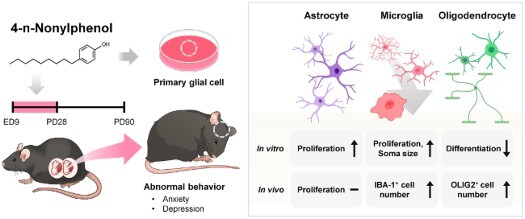

## Introduction

Endocrine-disrupting chemicals (EDCs) are exogenous substances that are widely pervasive in the environment and have the harmful potential to alter the functions of the endocrine system of organisms, leading to an increased risk of developing various diseases, including diabetes, hypothyroxinemia, and cancer (Casals-Casas and Desvergne 2011; Yilmaz et al. 2020; Guarnotta et al. 2022). Endocrine disruptors are utilized for the manufacturing of industrial products, including plasticizers and flame retardants, as well as in everyday products such as cosmetics, thermal paper, paints, and herbicides. These chemicals can also be detected in food and drinking water, thereby exposing humans to EDCs via various pathways (Kabir et al. 2015; Pironti et al. 2021; Ahn et al. 2022). 4-n-Nonylphenol (NP), a typical alkylphenol-type EDC, is a nonionic surfactant frequently found in personal care products, such as cosmetics and synthetic leather (Bhandari et al. 2021). Owing to its high versatility, long-term durability, and high lipophilicity, NP is recognized as a significant environmental pollutant because of its presence in the marine ecosystem, soil, sludge, and even within organisms, including humans (Ademollo et al. 2008; Vargas-Berrones et al. 2020). Recent research has revealed the existence of NP in human urine at a concentration of 0.46 µg/g creatinine and human breast milk at concentrations reaching up to 18.1 ng/mL in some cases, suggesting that humans are consistently exposed to NP (Sise and Uguz 2017; Xu et al. 2022).

Numerous studies have shown that NP has detrimental effects on various organs in humans and animals. NP exposure for 50 days remarkably decreased sperm density and testosterone levels and increased apoptosis in seminiferous tubules of adult male rats (Han et al. 2004). In another study, 4-week-old rats exposed to NP for 15 days displayed reduced thyroxine levels and dilation of the endocrine reticulum in thyroid follicular cells (Xi et al. 2013). Several studies have demonstrated that NP exposure adversely affects the brain health. In a previous study using adult rats exposed to NP for 35 days, NP accumulation in the hippocampus was linked to deficit in memory recognition and anxiety-like behavior (Kazemi et al. 2018). Chronic administration of NP to male rats resulted in downregulation of the brain-derived neurotrophic factor (BDNF)-TrkB-CREB signaling network in the hippocampus, contributing to anxiety-like behavior (Tang et al. 2022). Additionally, NP exposure during pregnancy can lead to its transfer to the fetus through the placenta and breast milk, potentially posing a risk to fetal brain health (Huang et al. 2014). Recent research has indicated that male offspring rats exposed to NP during neurodevelopmental stages exhibited autism spectrum disorder (ASD)-like behavioral symptoms, such as social deficit and repetitive behavior (You et al. 2023). However, there have been few reports on the effects of maternal NP exposure on brain development.

Glial cells in the central nervous system, mostly consisting of astrocytes, microglia, and oligodendrocyte lineage cells, provide structural support and aid neuronal functions by maintaining homeostasis in the microenvironment of the brain (Jäkel and Dimou 2017; Allen and Lyons 2018). In recent decades, research has increasingly emphasized the crucial role of glial cells in regulating brain functions, such as movement, mood, memory, and cognition, as well as their involvement in the development and progression of neurological diseases (Cotter et al. 2001; Vila et al. 2001; Dimou and Götz 2014; Sa et al. 2022). Recent studies have shown that the disruption of glial cell function by EDCs can result in behavioral impairments. Prenatal exposure to bisphenol A (BPA), a representative EDC, increased the number of microglia in the murine embryonic hypothalamus (Kundakovic et al. 2013). In addition, offspring mice exposed to BPA during lactation showed an increase in the microglia numbers in the prefrontal cortex and exhibited depression and anxiety-like behaviors (Wang et al. 2023). However, studies on the effects of NP exposure on glial cells are limited.

In this study, we aimed to investigate whether NP exposure during the neurodevelopmental period affects glial cells and behavior using primary mixed glial cells and male offspring mice to NP from embryonic day (ED) 9 to postnatal day (PD) 28. NP exposure heightened the proliferation of astrocytes and microglia in primary glial cells. Maternal NP exposure increased the number of microglia and oligodendrocytes in the cerebral cortex of offspring. Male offspring mice exposed to NP displayed anxiety– and depressive-like behaviors. Taken together, our findings show that maternal NP exposure has detrimental effects on the brain development and behavior in adulthood.

## Materials and methods

### Animals and chemical treatment

Eight-week-old C57BL/6J male and female mice (22–25 g) were obtained from Samtako (Osan, Gyeonggi, Republic of Korea). After a 1-week acclimatization period, female mice were mated overnight with male mice at a ratio of 2:1. The emergence of the vaginal plug was designated as ED0.5. Pregnant mice were individually housed and randomly divided into vehicle and NP exposure groups (*n* = 2 pregnant mice per group). To avoid sex differences, only male offspring were included in this study. From ED9 to PD28, maternal mice were administered corn oil daily (vehicle group) or NP (Alfa Aesar, Thermo Fisher, MA, USA) dissolved in corn oil at a dose of 50 mg/kg/day via subcutaneous injection (NP exposure group). After weaning on PD28, male offspring were separated into the vehicle and NP 50 mg/kg/day groups and raised until PD90. All mice used in this study were housed in cages at a constant temperature of 21 ± 1 °C, humidity of 40–60%, and 12 h/12 h light-dark cycle.

### Primary mixed glial cell culture

Primary mixed glial cell culture was performed as previously described with slight modifications (Chen et al. 2013). Whole brains were isolated from P1 mice, and the brain meninges were removed. Tissues were digested into single cells using trypsin (Welgene, Gyeongsan, Republic of Korea) and triturated. After being placed in poly-D-lysine (PDL)-coated T75 flasks, glial cells were cultured in Dulbecco’s modified Eagle’s medium (DMEM; Capricorn Scientific, Ebsdorfergrund, Germany) supplemented with 10% fetal bovine serum (FBS; Welgene) and 1% penicillin/streptomycin (P/S). The day of seeding was set as day 0 *in vitro* (DIV0). The culture medium was replaced on DIV5 in each flask, and the cells were incubated in an incubator with 5% CO_2_ at 37 °C for 10 days until approximately 90% confluency. For the 5-bromo-3-deoxyuridine (BrdU) proliferation assay of astrocytes, 5 × 10^4^ cells were plated in PDL-coated 24-well plates. On DIV1, microglial cells were incubated with 10 μM/mL BrdU (Sigma-Aldrich, St. Louis, MO, USA) at 37 °C after 12 h of NP treatment and harvested after 4 h of BrdU treatment. NP samples were dissolved in dimethyl sulfoxide (DMSO), and 0.1% DMSO solution was used as control.

### Isolation of microglia

As previously described, microglia were isolated from DIV10 primary mixed glial cell cultures (Tamashiro et al. 2012). Briefly, glial cells on DIV10 were agitated for 1 h at 180 rpm and 37 °C, and floating microglia cells were collected, centrifuged for 5 min at 500 ×* g*, and re-suspended in the same medium. The isolated microglial cells (1 × 10^5^/well) were plated into PDL-coated 24-well plates and cultured. On DIV1, microglial cells were treated with 10^−11^ M, 10^−8^ M, or 10^−5^ M NP to investigate its effects on microglial morphology and incubated for 48 h. For the proliferation assay, DIV1 microglial cells were treated with 10^−11^ M, 10^−8^ M, or 10^−5^ M concentration of NP and harvested after incubation with BrdU for 4 h.

### Isolation and differentiation of oligodendrocyte precursor cells

Primary oligodendrocyte precursor cells were isolated from primary mixed glial cells as previously described, with slight modifications (Chen et al. 2007). Briefly, suspended microglial cells were removed from DIV12 primary mixed glial cells by agitating the flasks for 1 h under the aforementioned conditions and washed twice with 1× Dulbecco’s phosphate-buffered saline (DPBS). To isolate oligodendrocyte precursor cells, primary glial cells were shaken for 4 h at 37 °C and 250 rpm, and cell suspension was collected. The cell pellet was suspended in DMEM (Capricorn Scientific) supplemented with 10% FBS (Welgene), B27, N2, 0.1% bovine serum albumin, 10 ng/mL platelet-derived growth factor-AA (Peprotech, NJ, USA), and 1% P/S. The oligodendrocyte precursor cells were plated into 24-well plates coated with PDL. For proliferation assay, DIV1 oligodendrocyte precursor cells were exposed to 10^−11^ M, 10^−8^ M, or 10^−5^ M NP following BrdU treatment as previously described. To investigate the effect of NP on oligodendrocyte morphology, the medium was replaced with a growth factor-free oligodendrocyte differentiation medium supplemented with NP on DIV3. Oligodendrocyte cells were harvested after incubation with NP for 48 h.

### Cell viability assay

Cell viability was analyzed using EZ-Cytox cell viability kit (DoGenBio, Seoul, Republic of Korea) according to the manufacturer’s protocol. Briefly, glial cells (5 × 10^4^/well) were seeded into a PDL-coated 96-well plate and incubated under 5% CO_2_ at 37 °C for 24 h. The cells were treated with NP at various concentrations (10^−13^ M, 10^−11^ M, 10^−9^ M, 10^−7^ M, 10^−5^ M, 10^−4^ M, 10^−3^ M, and 10^−2^ M). After 24 h, 10 μL of water-soluble tetrazolium salt solution was added to each well, and the cells were incubated for 1–2 h at 37 °C. Absorbance was measured at 450 nm using Epoch microplate spectrophotometer (BioTek, Winooski, VT, USA), and cell viability percentage was calculated by normalization to DMSO control.

### Immunofluorescence staining

Immunofluorescence staining was performed as described previously (Jung et al. 2017; Shin et al. 2023). The mice were anesthetized with Avertin (tert-amyl alcohol: 240486, Sigma-Aldrich; 2,2,2-tribromoethanol: T48402, Sigma-Aldrich) at a dose of 2.5%/g body weight. The brains were dissected and fixed in 4% paraformaldehyde at 4 °C. After 24 h of post-fixation, brains were transferred to 1× PBS and sectioned coronally at 60-μm thickness with a vibratome (SM2010R, Leica, Wetzlar, Germany). The brain sections were preserved in 1× PBS at 4 °C.

### Staining without BrdU

The glial cells and brain sections were fixed in 4% paraformaldehyde and permeabilized with PBS containing Triton X-100 (0.1% for cells and 0.5% for brain tissues). The glial cells or brain sections were blocked for 1 h using PBS with 5% donkey serum and 0.25% Triton X-100, followed by overnight incubation at 4 °C with the following primary antibodies: anti-GFAP (Abcam, Cambridge, UK; cat. no. ab53554, 1:500), anti-IBA-1 (Wako, VA, USA; cat. no. 019-19741, 1:500), anti-OLIG2 (Millipore, MA, USA; cat. no. AB9610, 1:500), and anti-MBP (Santa Cruz, CA, USA; cat. no. SC-271524, 1:500). For incubation with secondary antibodies, the cells or brain sections were incubated for 1 h with a secondary antibody solution containing Alexa Fluor 594 donkey anti-goat IgG (Invitrogen, Carlsbad, CA, USA; cat. no. A11055, 1:1000), Alexa Fluor 488 goat anti-rabbit IgG (Invitrogen; cat. no. A11034, 1:1000), Alexa Fluor 594 goat anti-rabbit IgG (Invitrogen; cat. no. A110112, 1:1000), Alexa Fluor 488 goat anti-mouse IgG (Invitrogen; cat. no. A11001, 1:1000), and Alexa Fluor 594 goat anti-mouse IgG (Invitrogen; cat. no. A110005, 1:1000) with 100 ng/mL 4’,6-diamidino-2-phenylindole (DAPI, Sigma-Aldrich). The cells or tissues were placed in Fluoro-Gel solution (Emsdiasum, Hatfield, PA, USA) and examined under a fluorescence microscope (Thunder Imager 3D Assay; Leica Microsystems).

### Staining with BrdU

Glial cells were permeabilized in PBS containing 0.1% Triton X-100. The cells were incubated in 1 M HCl (Daejung Chemicals & Metals Co., Gyeonggi-do, Republic of Korea) for 30 min, neutralized with 0.1 M boric acid (Sigma-Aldrich) for 30 min, and then blocked with PBS containing 5% donkey serum and 0.25% Triton X-100 for 30 min. For BrdU immunostaining, the cells were incubated with the corresponding primary antibodies and BrdU (BD Biosciences, NJ, USA; cat. no. 555627, 1:1000) overnight at 4 °C. The cells or tissues were incubated with secondary antibodies and mounted on Fluoro-Gel solution (Emsdiasum). The fluorescently labeled samples were observed using a fluorescence microscope (Thunder Imager 3D Assay; Leica Microsystems).

### Behavioral analysis

Mouse offspring were randomly selected for behavioral assessment at 7 and 10 weeks of age, as previously described (Shin et al. 2024). All behavioral tests were conducted during the light cycle. The behavioral testing room was maintained under the same and constant environmental conditions as during breeding. Additionally, external factors (noise, unnecessary light, and smell) were avoided to nullify the effect of stressful conditions on behavioral test results. On the testing days, the mice were moved to the testing room at least 30 min before starting the test. Behavioral tests were performed by laboratory technicians blinded to the mouse group information. A resting period of at least two days was provided between two consecutive tests. The experimental areas were sanitized with 70% ethanol before and after each test.

### Open field test

The open field test was performed in an acrylic cube (50 × 50 × 30 cm) with a white bottom and black wall, as previously described (So et al. 2023). Mice were placed individually near the wall, headed to the center, and allowed to move freely for 5 min. Mouse locomotor activity was recorded and analyzed using EthoVision XT16 software (Noldus, Leesburg, VA, USA). The time spent in the center zone (25 × 25 cm imaginary square), frequency of entering the center zone, and distance traveled were evaluated.

### Tail suspension test

The tail suspension test was performed according to a previously described method, with slight modifications (Moon et al. 2023). Mice were individually suspended on a shelf 50 cm above a table and allowed to move without restraint for 6 min. The recorded videos were analyzed using EthoVision XT16 software (Noldus) to determine the duration of immobility over the last 5 min.

### Forced swimming test

The forced swimming test followed a previously described protocol with minor adjustments (Shin et al. 2023). The mice were carefully placed in a glass cylinder (height: 20 cm, diameter: 15 cm) filled with water at a temperature of 23 ± 1 °C and a depth of 12 cm, and allowed to swim for 6 min. The duration of immobility during the last 5 min was recorded using a camera, and the videos were analyzed using EthoVision XT16 software (Noldus).

### Sucrose preference test

To investigate the impact of NP exposure on anhedonic behavior of mice, the sucrose preference test was performed according to a previously described protocol, with slight modifications (Serchov et al. 2016). Briefly, the animals were provided two sipper tubes overnight in each cage, each containing plain water and 5% sucrose solution. The bottles were weighed before being placed in the home cage at 16:00 and weighed again after removal at 09:00 the following day. The sucrose solution intake volume was measured by calculating the change in bottle weight. Relative sucrose preference was determined by dividing the volume of sucrose solution consumed by the total volume consumed by the mice.

### Statistical analysis

All data are presented as mean ± SEM and were analyzed using GraphPad Prism 8.0.1 (USA). Statistical significance was determined using two-tailed unpaired Student’s *t*-tests for two-group comparisons or one-way analysis of variance (ANOVA) followed by Bonferroni’s multiple comparison tests for multiple comparisons. *P* < 0.05 was regarded statistically significant and marked as *; *P* < 0.01, *P* < 0.005, and *P* < 0.001 were set as highly significant and marked as **, ***, and ****, respectively. *P* values for each comparison are shown in figure legends. All experiments were performed in a randomized and blinded manner. Experimental data from all treatment groups were compared with those from the vehicle groups. Each experiment was performed in triplicate.

## Results

### Exposure to NP at low doses increased cell proliferation of astrocytes in primary mixed glial cell

**Figure 1.**
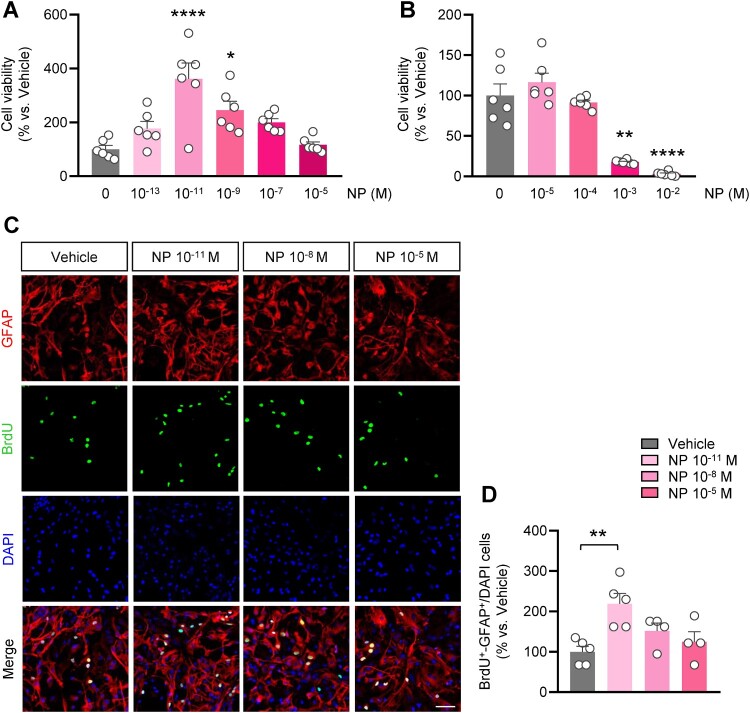
NP exposure increased the proliferation of primary mixed glial cells and astrocytes. **(A)** Cell viability assay was performed 24 h after treatment with increasing doses of NP. Growth of primary mixed glial cells was significantly increased following treatment with 10^−11^ M and 10^−8^ M NP [10^−11^ M NP: t_12_ = 6.023, *P* < 0.0001; 10^−9^ M NP: t_12_ = 3.338, *P* = 0.0113; *n* = 6 per group; two-tail Student’s *t*-test]. **(B)** Cell viability assay was used to investigate the cell viability at high doses of NP [10^−3^ M NP: t_12_ = 6.974, *P* < 0.0001; 10^−2^ M NP: t_12_ = 8.233, *P* < 0.0001; *n* = 6 per group; two-tail Student’s *t*-test]. **(C)** Representative image showing DIV1 primary mixed glial cells stained with GFAP (red) and BrdU (green). Scale bar = 50 μm. **(D)** Quantitative analysis for the proliferation rate of GFAP^+^ cells. The average BrdU^+^-GFAP^+^/DAPI percentage in the vehicle group was considered 100%. *n *= 3, cell culture was replicated three times [*F*_3,14 _= 6.211, *P* = 0.0066; one-way ANOVA with Bonferroni correction test]. Data are indicated as mean ± SEM. *P < 0.05, **P < 0.01, ****P < 0.0001 vs. vehicle.

First, we performed cell viability assays to investigate the toxicity of NP on primary mixed glial cells. After a 24-h incubation with NP at different concentrations, the viability of primary mixed glial cells was assessed. We observed that exposure to low doses of NP from 10^−11^ M to 10^−9^ M significantly increased cell viability (Figure 1A), whereas cells treated with NP at doses of 10^−3^ M and 10^−2^ M exhibited notable reduction in cell viability (Figure 1B). To investigate how different concentrations of NP affect each cell type of primary mixed glial cell, NP 10^−11^ M, 10^−8^ M, and 10^−5^ M were chosen as working concentrations. The majority of primary mixed glial cells (over 80%) are astrocytes, whereas approximately 20% are composed of microglia and oligodendrocyte precursor cells (Chen et al. 2013). To explore whether NP exposure increases astrocyte proliferation, primary mixed glial cells were exposed to NP and BrdU, followed by immunolabeling with glial fibrillary acidic protein (GFAP), an astrocyte-specific marker (Figure 1C). In primary mixed glial cells exposed to NP 10^−11^ M, the number of GFAP ^+ ^-BrdU^+^ cells was remarkably higher than that of the vehicle group, whereas no significant differences were found among the NP 10^−8^ M, NP 10^−5^ M, and vehicle groups (Figure 1D). These results indicate that a low dose of NP exposure elevated cell proliferation, particularly in astrocytes of primary mixed glial cells.

### NP exposure induced proliferation and morphological changes in microglia

**Figure 2.**
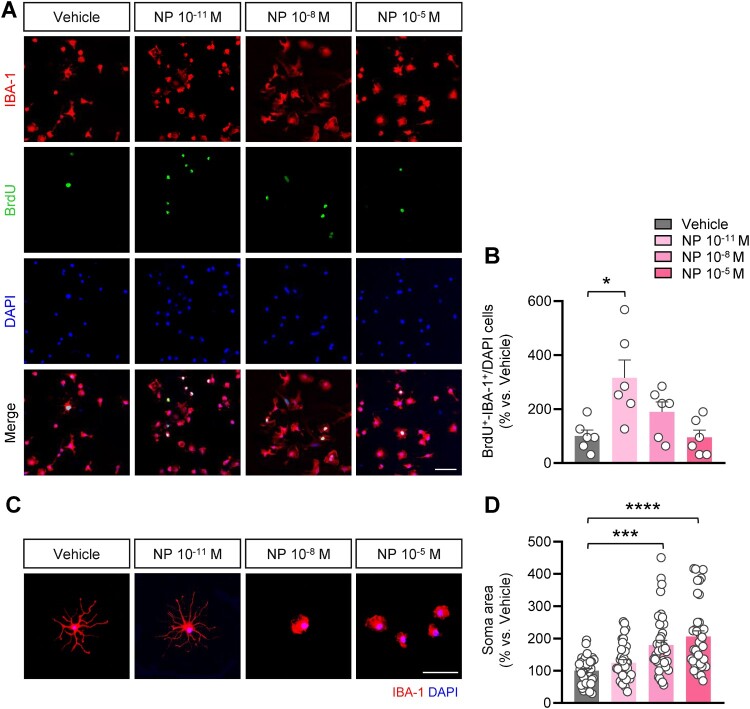
NP exposure increased the proliferation and soma size of primary microglial cells. **(A)** Primary microglial cells at DIV1 were immunostained using IBA-1 (red) and BrdU (green) antibodies. Scale bar = 50 μm. **(B)** Quantification of (A). The number of BrdU^+^-IBA-1^+^/DAPI-stained cells was significantly elevated after exposure to 10^−11^ M NP. The average BrdU^+^-IBA-1^+^/DAPI percentage in the vehicle group was considered 100%. *n *= 3, cell culture was replicated three times [*F*_3,20 _= 6.225, *P* = 0.0037; one-way ANOVA with Bonferroni correction test]. **(C)** Morphology of isolated microglia was examined by immunostaining using IBA-1 antibodies after treatment with NP for 48 h. Scale bar = 50 μm. **(D)** Exposure to 10^−8^ M NP significantly increased the area of microglial cell at DIV3. The average soma size of IBA-1^+^ cells in the vehicle group was considered 100%. *n *= 3, cell culture was replicated three times [*F*_3,148 _= 14.58, *P* < 0.0001; vehicle vs. 10^−8^ M NP: t_76_ = 4.393, *P* = 0.0001; vehicle vs. 10^−5^ M NP: t_76_ = 5.888, *P* < 0.0001; *n* = 38 per group; one-way ANOVA with Bonferroni correction test]. Data are indicated as mean ± SEM. *P < 0.05 vs. vehicle.

To examine whether exposure to NP affects the proliferation of microglial cells, we treated microglial cells at DIV1 with NP and BrdU and immunolabeled them with ionized calcium-binding adapter molecule 1 (IBA-1), a microglia-specific marker, after 4 h (Figure 2A). In isolated microglial cells treated with NP 10^−11^ M, there was a significant increase in the number of IBA-1^+^-BrdU^+^ cells compared with the vehicle group. However, the number of IBA-1^+^-BrdU^+^ cells exposed to NP concentrations of 10^−8^ M and NP 10^−5^ M was comparable to that observed in the vehicle group (Figure 2B). Microglia can be activated by extracellular stimuli, leading to structural and functional changes, including inflammatory responses (Vidal-Itriago et al. 2022). Representative features of activated microglia include soma enlargement and process retraction (Davis et al. 2017). To assess the influence of NP exposure on microglial morphology, the isolated microglial cells incubated with NP for 48 h were labeled with IBA-1, and the soma size of IBA-1^+^ cells was quantified (Figure 2C). We found that exposure to NP at doses of 10^−8^ M and 10^−5^ M significantly increased the soma area compared with that of the vehicle group, whereas no changes were observed in microglial cells exposed to NP 10^−11^ M (Figure 2D). These findings suggest that NP exposure induces microglial proliferation at low doses and triggers microglial activation at higher concentrations.

### Impact of NP on the proliferation and differentiation of oligodendrocytes

**Figure 3.**
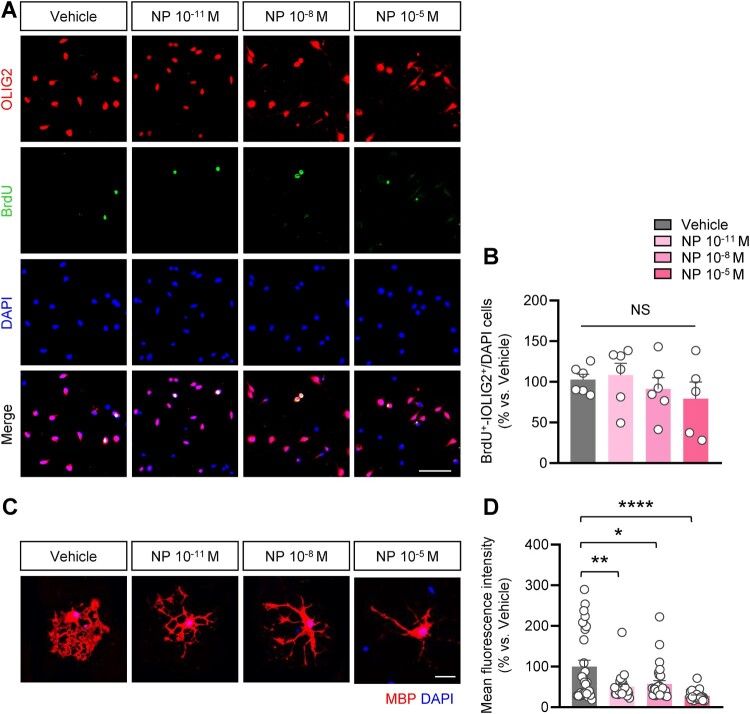
NP exposure altered the morphology of primary oligodendrocyte cells. **(A)** DIV1 primary oligodendrocyte precursor cells were immunostained using OLIG2 (red) and BrdU (green) antibodies. Scale bar = 50 μm. **(B)** Quantitative analysis was conducted to investigate the number of BrdU^+^-OLIG2^+^/DAPI-stained cells after NP exposure. The average BrdU^+^-OLIG2^+^/DAPI percentage in the vehicle group was considered 100%. *n *= 3, cell culture was replicated three times. **(C)** After induction of differentiation of oligodendrocyte, the morphology of oligodendrocyte was examined by immunostaining using MBP antibodies after treatment with NP for 48 h. Scale bar = 10 μm. **(D)** Mean fluorescence intensity was decreased in primary oligodendrocyte cells after NP treatment. The average MBP^+^ cells in the vehicle group was considered 100%. *n *= 3, cell culture was replicated three times [*F*_3,112 _= 10.15, *P* < 0.0001; vehicle vs. 10^−11^ M NP: t_58_ = 3.717, *P* = 0.0019; vehicle vs. 10^−8^ M NP: t_58_ = 0.0111, *P* = 0.0001; vehicle vs. 10^−5^ M NP: t_58_ = 5.386, *P* < 0.0001; *n* = 29 per group; one-way ANOVA with Bonferroni correction test]. Data are indicated as mean ± SEM. *P < 0.05 vs. vehicle.

To determine whether NP exposure affects the proliferation of oligodendrocyte precursor cells, these cells were separated from primary mixed glial cultures and incubated with different concentrations of NP. After 4 h of BrdU incubation, the cells were labeled with oligodendrocyte transcription factor 2 (OLIG2), an oligodendrocyte-specific marker expressed throughout all stages of oligodendrocyte differentiation (Zhang et al. 2022). We found that no differences were observed in the number of OLIG2^+^-BrdU^+^ cells between NP-exposed cells and vehicle-exposed cells (Figure 3A, B). Next, to explore the influence of NP exposure on oligodendrocyte differentiation, the cells were incubated in a growth factor-free medium supplemented with NP for 48 h. Differentiation and maturation of oligodendrocytes begin in the absence of growth factors (Hu et al. 2008). Mature oligodendrocytes possess the ability to myelinate, extend their membranes to create insulating sheaths around the axons, and express myelin basic protein (MBP) (Barateiro and Fernandes 2014). In NP-treated oligodendrocytes, the mean fluorescence intensity of MBP^+^ cells was significantly lower than that of vehicle-treated cells, suggesting that NP exposure disrupts the myelination of oligodendrocytes during lineage progression (Figure 3C, D). These results suggest that NP exposure interferes with oligodendrocyte differentiation, but not with proliferation.

### NP exposure elevated the number of microglia and oligodendrocytes in the cortex of offspring brain

**Figure 4.**
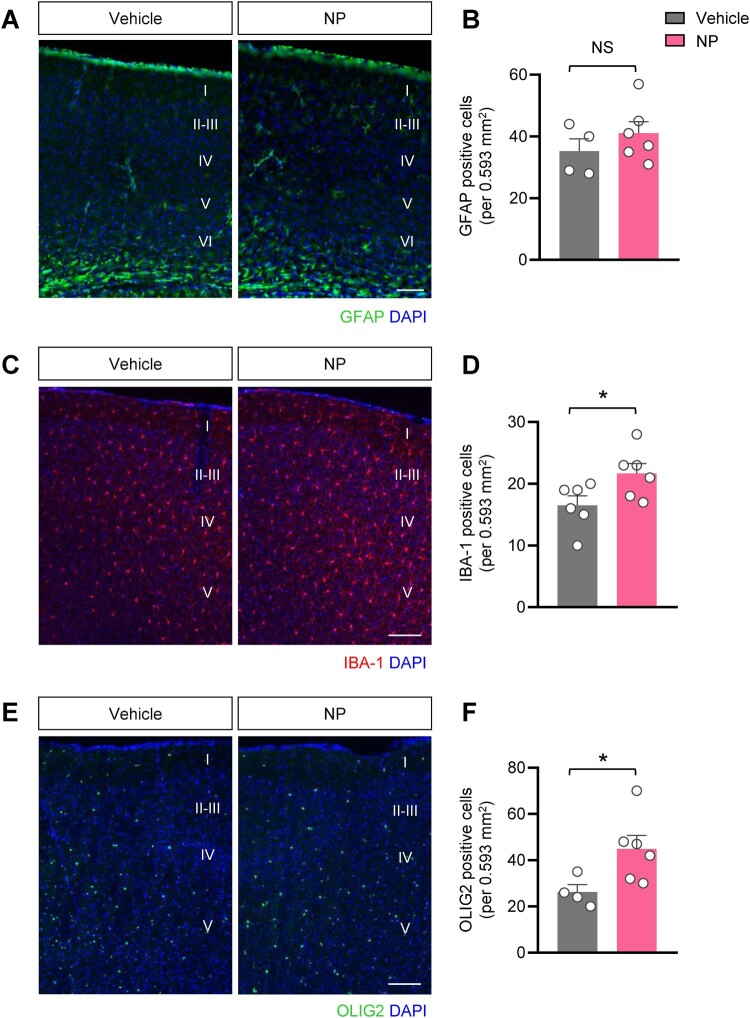
Maternal NP exposure increased the number of microglia and oligodendrocyte cells in the cortex of adult offspring mice. **(A)** Representative fluorescent images of the brain cortex immunostained with the astrocyte marker GFAP (green). Scale bar = 100 μm. **(B)** Bar graphs displayed no difference in the number of GFAP^+^ cells between vehicle – and NP-treated offspring brains. **(C)** Representative images showing the cortex of adult offspring mice stained with the microglia marker IBA-1 (red). Scale bar = 100 μm. **(D)** Quantitative analysis showed that the number of IBA-1^+^ cells was significantly increased in the NP-treated group [t_8_ = 2.320, *P* = 0.0428; two-tailed Student’s *t* test]. **(E)** The cerebral cortex of adult offspring exposed to NP was labeled with the oligodendrocyte marker OLIG2 (green). Scale bar = 100 μm. **(F)** Quantitative analysis of immunolabeled cells showed that NP exposure significantly increased the number of OLIG2^+^ cells [t_8_ = 2.389, *P* = 0.0439; two-tailed Student’s *t* test]. 0.593 mm^2^ image of cortical layer from the brain of each PD90 offspring. *n *= 4 for vehicle group and *n *= 6 for NP group. Data are indicated as mean ± SEM. NS means no significance. *P < 0.05 vs. vehicle.

To examine the effects of NP exposure on glial cells during brain development *in vivo*, we treated pregnant mice with NP 50 mg/kg/day during pregnancy and lactation. We evaluated the number of glial-specific marker-positive cells in the cerebral cortex of PD90 offspring. There was no substantial difference in the number of GFAP^+^ cells between the vehicle – and NP-treated groups (Figure 4A, B). However, we observed that the number of IBA-1^+^ cells significantly increased in the cortex of mice in the NP-treated group (Figure 4C, D). In addition, compared with the vehicle group, NP-treated offspring displayed a notable increase in the number of OLIG2^+^ cells in the cerebral cortex (Figure 4E, F). These findings suggest that maternal exposure to NP upregulated the distribution of microglia and oligodendrocytes in adult offspring mice.

### Maternal NP exposure induced anxiety- and depressive-like behaviors in adult offspring mice

**Figure 5.**
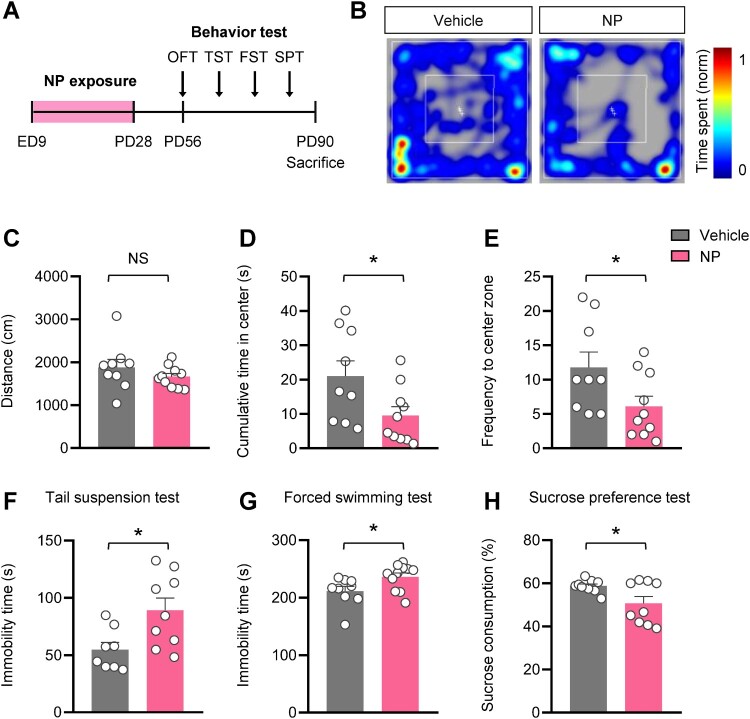
Adult offspring mice exposed to NP during neurodevelopment displayed anxiety – and depressive-like behavior. **(A)** Schematic experimental design including schedules for NP treatment and behavioral assessment. Maternal mice were administered NP during gestation and lactation. **(B)** Representative heatmap images showing the movement of mice in the open field test. **(C)** Distance traveled in an open field. **(D)** Cumulative time spent in the center and **(E)** frequency of entering the center zone were decreased in the NP-treated group [cumulative time in the center: t_17_ = 2.273, *P* = 0.0363; frequency for entering the center zone: t_17_ = 2.176, *P* = 0.0440; *n *= 9 for vehicle group and *n *= 10 for NP group; two-tailed Student’s *t* test]. **(F)** Tail suspension test [t_15_ = 2.690, *P* = 0.0168; *n *= 8 for vehicle group and *n *= 9 for NP group; two-tailed Student’s *t* test]. **(G)** Forced swimming test [t_18_ = 2.343, *P* = 0.0308; *n *= 9 for vehicle group and *n *= 11 for NP group; two-tailed Student’s *t* test]. **(H)** Sucrose preference test [t_16_ = 2.376, *P* = 0.0303; *n *= 8 for vehicle group and *n *= 8 for NP group; two-tailed Student’s *t* test]. Data are indicated as mean ± SEM and representative of triplicate experiments. NS means no significance. *P < 0.05 vs. vehicle.

To explore whether NP exposure during gestation and lactation induces abnormal behavior in adult offspring, we conducted several behavioral tests according to the experimental schedule shown in Figure 5A. First, we performed an open-field test to examine anxiety-like behavior in offspring mice. Anxiety-like behavior was analyzed by measuring the total movement distance, cumulative duration in the center area, and frequency of entering the center during the test. There was no significant difference in the distance traveled between the vehicle – and NP-treated groups (Figure 5B, C). However, the cumulative time spent and frequency of entering the center remarkably decreased in the NP-treated offspring mice (Figure 5D, E). These results imply that adult offspring mice-exposed to NP during neurodevelopment displayed anxiety-like behavior. Next, tail suspension and forced swimming tests were carried out to investigate whether NP exposure led to despair-like depressive behavior in mice. The tail suspension test results showed a notably longer immobility time in NP-exposed mice than in the vehicle group (Figure 5F). Similarly, significantly longer immobility time was observed in mice exposed to NP than in the vehicle group in the forced swimming test (Figure 5G). Additionally, we conducted the sucrose preference test to examine whether NP exposure induces anhedonia-like depressive behavior in mice (Liu et al. 2018). We observed that the NP-exposed mice consumed significantly less sucrose water than those in the vehicle group (Figure 5H). These findings suggest that maternal exposure to NP may trigger anxiety – and depressive-like behaviors in adult offspring mice.

## Discussion

Exposure to EDCs during early brain development increases the risk of neurodevelopmental disorders (Masuo and Ishido 2011). The present study demonstrates that NP exposure during pregnancy and lactation affects the proliferation and morphology of glial cells, leading to anxiety – and depressive-like behaviors in adult male offspring mice. As previously reported, the NP dose of 50 mg/kg/day used in this study was below the no-observed-adverse-effect level for maternal toxicity (EPA 2010). The administration of 50 mg/kg/day NP to mice is equivalent to 4.05 mg/kg/day in a human dose, as determined using the body surface area normalization method recommended in the US Food and Drug Administration current guidelines (Reigner and Blesch 2002; Nair and Jacob 2016). Previous studies using a physiological pharmacokinetic model have demonstrated that humans can be exposed to NP at doses higher than 4.05 mg/kg/day (Jeong et al. 2022). Given the longer gestation and lactation periods in humans than in mice, administering 50 mg/kg/day NP to mice is presumed to indirectly represent the physical impact of NP on the health of human offspring.

We first reported that low concentrations of NP drastically increased astrocyte proliferation in primary mixed glial cells, and that maternal exposure to NP 50 mg/kg/day did not affect the distribution of astrocytes in the cortex of offspring mice. The discrepancy between *in vitro* and *in vivo* results may be attributed to differences in NP administration methods, exposure duration, and absorption rates within the body (Green et al. 2003; Algharably et al. 2022). Few studies have reported the effects of NP on astrocytes. A previous study reported that adult rats exposed to 100 mg/kg/day NP for 21 days by oral gavage exhibited an increased number of astrocytes in the cerebral cortex (Ceylan et al. 2023). This study has limitations in that the effects of different doses of NP on astrocytes during neurodevelopment have not been sufficiently explored compared with the effects of various concentrations of NP on astrocyte proliferation *in vitro*. Further studies using animal models are required to investigate the effects of various NP concentrations on astrocytes during brain development.

In this study, NP exposure increased the proliferation of microglia in primary microglial cells and in the cerebral cortex of offspring. Moreover, NP altered the morphology of microglia into an amoeboid form, characterized by an enlargement of soma size and dendritic retraction, implying that microglial activation was induced by NP. Previous studies have demonstrated that exposure to 2 mg/kg/day NP for 35 days can induce oxidative stress and increase the inflammatory response by accumulating reactive oxygen species in the hippocampus and amygdala of adult rats (Kazemi et al. 2018). An increasing number of studies indicate that aberrant activation and impairment of microglia, both in the developing and adult brain, are associated with the onset of mood disorders, such as anxiety and depressive behavior (Elmore et al. 2015; Nelson and Lenz 2017; Schaafsma et al. 2017; Park and Jung 2022). Maternal exposure to NP at a dose of 50 mg/kg/day elevated inflammatory cytokine levels, accompanied by an increase in microglial numbers in the hippocampus of PD7 and PD21 offspring mice (Gu et al. 2018; Qiu et al. 2019). These findings corroborate our results showing that maternal NP exposure increased the number of microglia in the cerebral cortex of adult offspring mice. Owing to the limitation of current studies that do not address the impact of NP exposure on the inflammatory response in the cortex of adult offspring mice, future work will focus on how NP influences inflammatory responses.

Myelin is generated by mature oligodendrocyte precursor cells (Simons and Nave 2015). The impairment of oligodendrocyte differentiation results in delayed myelination (Kuhn et al. 2019). We found that NP exposure decreased the expression of MBP, which is essential in myelination, in primary oligodendrocytes and elevated the number of oligodendrocytes in the cortex of adult offspring. These findings correspond with those of previous research that reported that maternal exposure to 50 mg/kg/day NP suspended myelination of the cerebellum in rat offspring (Jiang et al. 2020). Additionally, a transcriptional factor OLIG2 has a repressive role in oligodendrocyte differentiation and inhibition of myelinogenesis, which is consistent with our results on an increase of OLIG2^+^ cell number induced by NP (Zhang et al. 2022). We conclude that maternal exposure to NP impairs myelination in the brains of adult offspring.

Several studies have documented the effects of NP on brain health. A remarkable reduction in BDNF levels was observed in the hippocampus of adult rats administered 40 mg/kg/day NP for 90 days via oral gavage (Tang et al. 2022). Furthermore, the BDNF-TrkB signaling pathway was disrupted in the cerebellum of offspring mice exposed to NP from gestation through lactation, resulting in impaired dendritic growth of Purkinje cells (You et al. 2018). BDNF is extensively present in both neuronal and glial cells and is crucial for regulating neuroplasticity during brain development (Bathina and Das 2015). A previous study using primary astrocytes demonstrated that BDNF treatment significantly enhanced cell viability and promoted cell migration (Adam et al. 2023). Another study revealed that BDNF ameliorated the neuroinflammatory responses induced by lipopolysaccharides in cultured primary microglia (Charlton et al. 2023). BDNF exposure increased the number of MBP^+^ cells in basal forebrain-derived primary oligodendrocytes but not in cortical-derived primary oligodendrocytes (Du et al. 2003). These results support the presumption of an indirect relationship between NP-induced decreased BDNF expression and the impact of NP on glial cells observed in this study.

Numerous reports have shown that NP exposure triggered abnormal behaviors in animal models. Male zebrafish exposed to 100 μg/L NP for 50 days showed a notable decrease in aggressive behavior and locomotor activity (Xia et al. 2010). Sub-chronic NP exposure at a low dose of 4 mg/kg/day for 90 days induced anxiety behavior in adult male rats (Li et al. 2021). Few studies have investigated the effects of NP exposure during neurodevelopment on animal behavior. Previous studies using rats have indicated that maternal exposure to 50 mg/kg/day NP led to anxiety, social deficits, and stereotyped behaviors similar to the symptoms of ASD in offspring mice (You et al. 2023). In this study, we confirmed that maternal NP exposure can also result in despair – and anhedonia-like depressive behaviors in adult offspring mice using the tail suspension test, forced swimming test, and sucrose preference test, respectively. The modulation of synaptic plasticity during neurodevelopment is pivotal in determining an organism’s behavior (Kolb and Gibb 2011). Recent studies have revealed that NP exposure impairs synaptic plasticity, resulting in various behavioral disorders. Exposure to 4 mg/kg/day NP for 90 days significantly reduced the number of neurons, length and number of dendritic spines, and thickness of postsynaptic density, resulting in depressive behavior in adult rats (Yu et al. 2019). Rats exposed to 80 mg/kg/day NP for 90 days exhibited deficits in spatial memory and recognition, linked to the reduced expression of N-methyl-D-aspartate receptors and synapse-associated proteins in the hippocampus (Fu et al. 2023). Most studies on the toxicological effects of NP on brain health have primarily emphasized neuronal function. However, given the substantial role of glial cells in synaptic plasticity, further studies are required to investigate the association between the molecular impact of NP on glial cells and behavioral alterations.

## Conclusions

Our results demonstrated that NP exposure increased the proliferation of astrocytes and microglia in primary glial cell cultures. NP exposure triggered microglial activation and impaired myelination of oligodendrocytes. Moreover, maternal NP exposure increased the number of microglia and oligodendrocytes in the cerebral cortex of adult mice. NP-exposed mice exhibited anxiety – and depressive-like behaviors. This study highlights the detrimental effects of NP exposure during the early neurodevelopmental period on brain health.

## Data Availability

The data that support the findings of this study are available from the corresponding author, E.-M. J., upon reasonable request.
